# Determining and interpreting correlations in lipidomic networks found in glioblastoma cells

**DOI:** 10.1186/1752-0509-4-126

**Published:** 2010-09-07

**Authors:** Robert Görke, Anke Meyer-Bäse, Dorothea Wagner, Huan He, Mark R Emmett, Charles A Conrad

**Affiliations:** 1 Department of Computer Science, Karlsruhe Institute of Technology, Karlsruhe D-76128, Germany; 2 Department of Scientific Computing, Florida State University, Tallahassee, Florida 32310-4120, USA; 3 Ion Cyclotron Resonance Program, National High Magnetic Field Laboratory, Florida State University, Tallahassee, Florida 32310-4005, USA; 4 Department of Chemistry and Biochemistry, Florida State University, Tallahassee, Florida 32306, USA; 5 The University of Texas M. D. Anderson Cancer Center, Houston, TX 77030, USA

## Abstract

**Background:**

Intelligent and multitiered quantitative analysis of biological systems rapidly evolves to a key technique in studying biomolecular cancer aspects. Newly emerging advances in both measurement as well as bio-inspired computational techniques have facilitated the development of lipidomics technologies and offer an excellent opportunity to understand regulation at the molecular level in many diseases.

**Results:**

We present computational approaches to study the response of glioblastoma U87 cells to gene- and chemo-therapy. To identify distinct biomarkers and differences in therapeutic outcomes, we develop a novel technique based on graph-clustering. This technique facilitates the exploration and visualization of co-regulations in glioblastoma lipid profiling data. We investigate the changes in the correlation networks for different therapies and study the success of novel gene therapies targeting aggressive glioblastoma.

**Conclusions:**

The novel computational paradigm provides unique "fingerprints" by revealing the intricate interactions at the lipidome level in glioblastoma U87 cells with induced apoptosis (programmed cell death) and thus opens a new window to biomedical frontiers.

## Background

Glioblastoma are highly invasive brain tumors. The prognosis for patients with glioblastoma depends on many factors, including age, performance status, and histology grade of the glial neoplasm. The medial survival is approximately 14 months with maximal therapy. Glioblastoma are difficult to treat due to the resistance to conventional therapies as well as the ability to diffuse throughout the brain. Therefore, analytical description of tumor growth and response to therapeutic modalities, such as radiation and chemotherapy, has been a central research topic. Modeling of the tumor responding to chemotherapy is mostly of pharmacokinetic nature [1]. Other mathematical models are based on a conservation equation describing a modality of how to measure the growth of an infiltrating glioma [2]: the rate of change of tumor cell population equals the diffusion (motility) of tumor cells plus the net proliferation of those. Cell death is introduced as a loss term in [3]. A model combining patient's imaging, histopathologic and pharmacodynamic/genetic data when treated with temozolomide is presented in [4].

Interesting aspects of glial cell biology have recently been uncovered in laboratories evaluating the tumor suppressor protein wild type 53 (wt p53) [5,6]. It is well established that transfecting glioma cells with wild-type tumor protein p53 will trigger brisk apoptosis if the cell line harbors mutant p53, while the same transfection to cell lines which harbor the wt p53 will result in a reduction or elimination of invasion and motility [7]. A glioma cell line that harbors the wild-type form of the tumor suppressor protein p53 can be sensitized to undergo apoptosis by the addition of wt p53 along with chemotherapy (such as SN38) [5,6]. Recently, new insights into the pathobiology of glioblastoma cells have been obtained at the M.D. Anderson Cancer Center in Houston, Texas: transfer of the p53 gene by use of an adenovirus vector (Ad-p53) may be clinically applicable in human gliomas. Furthermore, it has been demonstrated that combined adenovirus transfection of wild-type p53 (wt p53) into glioma cells followed with chemotherapy treatment SN-38 may act to convert gliomas to an "apoptosis-sensitive" phenotype [8]. Moreover, wt p53 containing tumor cells, such as U87 MG will show reduced mobility and decreased invasion in matrigel motility assays after wt p53 gene therapy. A proteomic approach identified proteins that were involved in a phenotypic change in the high-grade glioma cell line U87 MG under the influence of transfection with wild-type p53 and additional cytotoxic chemotherapy with SN-38 [6]. This study showed that the expression of the protein galectin-1 is associated with malignancy and poor prognosis. The results suggest that galectin-1 is a relevant therapeutic target to downregulate in a clinical pharmacological setting to improve overall survival of high-grade glioma patients. Our current understanding of proteins such as galectin-1, interactions and pathways is detailed, yet it is still incomplete. Galectin 1 binds free beta-galactose residues on both glycoproteins and gangliosides (GM1 and asialo-GM1 gangliosides are known to be galectin-1 ligands) [9]. Gangliosides were first discovered by the German scientist Ernst Klenk in 1942. They are cell-type specific antigens that provide cell membrane structure, and play key roles in control growth, cell differentiation and cell/cell interactions. Gangliosides are implemented in different cancer types (such as glioblastoma) since some typical gangliosides are present in tumors, but are absent in normal healthy tissue [10].

Although connections between cancer and glycobiology have been described, the detailed chemical analysis of polar lipids has been problematic due to structural complexity as well as limitation of analytical techniques. Recently, He et al. [11] pioneered new analytical methodology with nano-liquid chromatography (nano-LC) separation followed by high mass accuracy and high mass-resolving power Fourier transform ion cyclotron resonance (FT-ICR) mass spectrometry (MS) analysis at 14.5T [12]. MS is useful in dealing with complex mixtures since the high mass resolution (narrow peak width) allows the signals of two ions of similar mass-to-charge ratio (m/z) to be detected as distinct ions. This new methodology has opened a new field of polar lipid profiling. A typical biological analysis from 2 million cells results in the identification of 600-800 different polar lipids of various polar heads and variable size and modified (degree of saturation and hydroxylation) nonpolar tails. Relative quantification of each polar lipid between treated and untreated cells results in highly complex, information rich data sets. The ultimate goal of this research is to link the changes in the polar lipids to the enzymes that control their synthesis/modifications/degradation (gene arrays and functional proteomics), which will provide a systems biology approach to the study of glioblastoma. The data generated will be used to drive logical, hypothesis driven experiments in the search for new therapeutic targets in glioblastoma research.

Since the lipid profiling methodology generates comparative semi-quantitative data on hundreds of different polar lipids with each experiment, the data analysis has become a daunting task. Novel approaches [13-15] are now needed to compile the existing biological lipid profiling experimental data and to maximize the information extraction from these databases. Once correlations in the lipid database have been identified, the reduced data will be correlated to the gene array data and proteomic data sets. This manuscript describes a novel algorithm for identification of correlations in the complex lipid data sets derived from gene treated and empty vector treated U87 glioblastoma cell lines. We research, implement and test graph clustering techniques as an equivalent to standard correlation networks. This novel approach combines analysis and visualization with the correlation structure of the lipid profiling data. This novel technique enables a better understanding and a mathematical analysis of the observed experimental results.

## Results and Discussion

In this paper we develop a novel paradigm to replace correlation networks standardly employed for displaying the topological properties of networks [16,17]. Traditional correlation networks are based on building a graph and determining the correlation scores between the nodes of the graph and then compare those values with a given threshold. For those exceeding the threshold an undirected edge is linked between the nodes. As a novel concept in this paper, we research, implement and test graph clustering techniques as an equivalent to standard correlation networks for the analysis of therapeutic outcomes for U87 MG glioblastoma cells.

**Figure 1:**
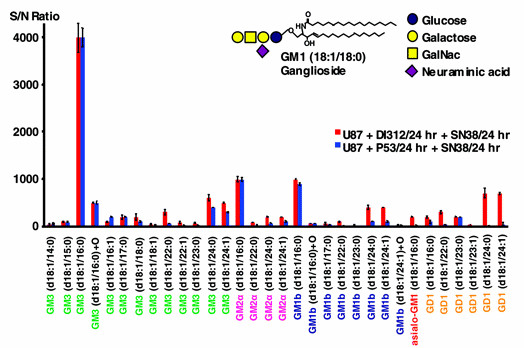
**Gangliosides profiles for the two treatments U87+DI312/24hr+SN38/24hr and U87+p53/24hr+SN38/24hr**. Ganglioside profiles for the polar lipid fraction of extracts of U87 cells following two different treatments. The effect of treatment with wild-type wt p53, which lowers the abundance of longer chain ceramides, is most pronounced for the GM1b and GD1 gangliosides. Figure extracted from [11] and awaiting copyright permission.

**Figure 2:**
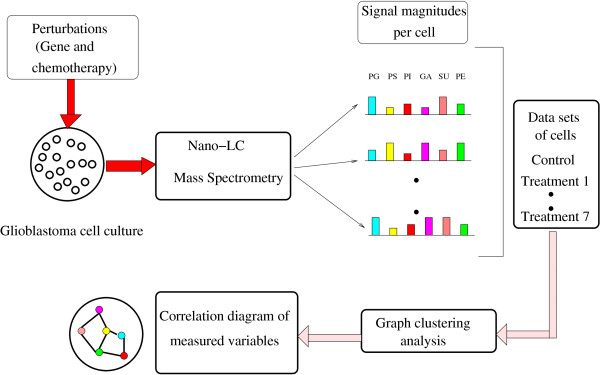
**Lipidomic platform data processing**. Graph clustering modeling with U87 MG glioblastoma cell data. The schematic shows a resulting correlation based on nano-LC/MS data. Seven different treatments are applied to a cell line. Graph clustering analysis is performed on this data and a correlation diagram reflecting dependencies in the underlying network is obtained. Abbreviations. PG means phosphatidylglycerol, PS means phosphatidylserine, PI means phosphatidylinositol, PE means phosphatidylethanolamine, GA means gangliosides and SU means sulfatide.

Global multivariate approaches such as the proposed novel graph clustering correlation network are needed in order to understand the potential disregulation of normal cellular responses in disease and their response to various therapeutic interventions. Moreover, because nano-LC/MS can find and assign hundred of lipids from glioma cells, it is possible to identify causal influence relationships involving multiple lipids. Building upon lipid measurements such as those illustrated in Figure 1 for the group of gangliosides for two treatments (here, treatments one and two listed in Table 1), Figure 2 shows the schematic of the graph clustering network based on multidimensional lipidomic data. The correlation structure is explored by varying the threshold between different lipids and the important changes between treatments by clusters of nodes of different sizes and fan-out degrees.

**Table 1:** Treatment conditions of U87 Glioblastoma Cell Cultures.

Treatment conditions of U87 Glioblastoma Cell Cultures.	
**Condition**	**Abbreviation**

Treatment 1	U87 + DI312/24h + SN38/24h

Treatment 2	U87 + p53/24h + SN38/24h

Treatment 3	U87 + SN38/24h + DI312/24h

Treatment 4	U87 + SN38/24h + p53/24h

Treatment 5	U87 + DI312

Treatment 6	U87 + p53/24h

Treatment 7	U87 + SN38/24h

Control	U87 cell only

### A Graph Model based on Correlation Networks

The notion of networks has recently started to permeate diverse fields of science as well as numerous concepts in our everyday lives. This development roots particularly strong in social network analysis, an area which spawned many concepts and methods that today experience a renaissance empowered by the ubiquity and availability of social networks, especially in the internet (another, technical network). The driving force behind the rise of this concept is the fact that a network is a very apt and well-perceivable representation of the interrelation of things. For this reason, we advocate the use of network analysis [18] in the field of lipidomics. Generally speaking, any set of interrelated entities constitutes a network, and can thus be modeled as a graph, which is the mathematical formalization of the term network. The evidence that lipids are related in their reaction to treatments leads to the fact that exploration and visualization of co-regulations in glioblastoma data can be done based on correlation network analysis. Such networks describe all dependencies between the domain variables. More precisely we define the simple (neither self-loops nor parallel edges are allowed), undirected (edges represent a mutual relationship) and weighted graph *G *= (*V*, *E*, *ω*) as follows. The set *V *of nodes is the set of all measured lipids while the set *E *of edges (i.e nodes pairs) describes the dependencies between these lipids by a weight function *ω *: *E *→ [0, 1] which expresses the strength of the dependencies. In our case *ω *is based on a matrix of correlation coefficients which is computed based on the pair-wise correlation between the concentrations of lipids in a given sample. Thus, for an edge *e *= {*u, v*}, the weight *ω *(*e*) is a measure of correlation, i.e., similarity between lipids *u *and *v*, with a value of 1 indicating identical measurements *x *and *y*, and values close to 0 indicating very different measurements. We base our correlation matrix on measured values *x *normalized with the standard normalization function *f*(*x*) = log(*x*+1). However, since this proved to be too non-discriminative for network analysis techniques, we actually used *f′*(*x*) = (*f*(*x*))^3^. The correlation of two measurements *x *and *y *is then computed as cor(*x*, *y*) = min{*f′*(*x*), *f′*(*y*)}/max{*f′*(*x*), *f′*(*y*)}, with the special case of cor(*x*, *y*) = 1 if *x *= *y *= 0.

**Figure 3:**
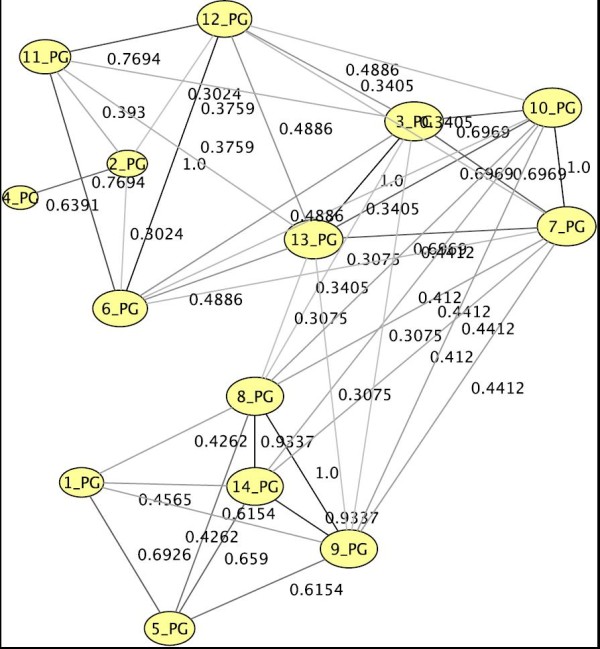
**Example of a correlation network for phosphatidylglycerols for U87+SN38/24hr**. A correlation network of the group of phosphatidylglycerols wrt. treatment 7 (see Tab. 1), based on a threshold of 0.3.

Since in this work we divide our studies up into different groups of lipids, i.e., gangliosides, phosphatidylinositol and phosphatidylglycerol, we construct three different base-networks for our exploratory clusterings, one for each group. Actual lipid correlation graphs are then obtained by choosing a treatment and a threshold value for the correlation coefficient values, i.e., edges with insignificant weights are pruned. Two nodes *u *and *v *that are connected by an edge are called *adjacent*, and an edge *e *and an end node *v *of *e *are called *incident*, abbreviated by *u *~ *v *and *e *~ *v*, respectively. Figure 3 is the network of phosphatidylglycerols. For the sake of readability we do not state full names of lipids in our graphs, but instead denote the index of the lipid in the corresponding chart (as, e.g., in Figure 3 for phosphatidylglycerols). The layout (i.e., the positions of nodes in a drawing) is determined with so called *force-directed *graph-drawing techniques. Roughly speaking, strong ties draw nodes together and weak ties let them drift apart. Heavier edges are darker than lighter edges and larger nodes share many heavy edges with other nodes. We can now identify groups of lipids that exhibit a similar behavior.

### Graph Clustering on Correlation Networks

We propose and tested a novel concept for analyzing correlation networks based on graph clustering. In Figure 3 we can easily perceive three rather densely knit groups of lipids, which share mostly weak links (if at all) to the other groups. Such a dense subset indicates that these lipids correlate strongly with each other wrt. to their reaction to the applied treatment, i.e., it is a cluster of similarly behaving lipids. While in a single small example this might be done by hand, this task is usually realized with *graph clustering *[18,19] algorithms, which automatically identify a clustering. Clustering methods discover natural concentration and large-scale inhomogeneities in relational data. A clustering C(G) of a graph *G *= (*V*, *E*) is a partition $C=\left\{C_{1},...,C_{k}\right\}$ of the set *V *of nodes into clusters *C_i _*(remember that a partition ℬ, = {*B*_1_, ..., *B_k_*} of a set *A *always guarantees $\cup_{i=1}^{k}B_{i}=A$ and *B*_*i *_∩ *B*_*j *_= ∅, ∀ *i *≠ *j *∈ {1, ..., *k*}).

Clusterings always follow the paradigm of intra-cluster density vs. inter-cluster sparsity. In other words, the edge-connectivity inside clusters is to be strong, while between clusters there should only be few, or light-weighted edges. In our context this means that the pairwise correlations of nodes inside one cluster are all rather high, and those of nodes in different clusters are comparably low. It is important to note that the number of clusters to be found is not preset by a parameter, instead it is determined by the algorithm and the instance. Since edge weights represent the strength of ties, our graph clustering algorithm takes these edge weights into account, which yields a higher precision than pruning light edges and then employing an un weighted algorithm. For the sake of readability, we still use the pruning only for our drawings.

For actual clustering tasks, the informal notion of intra-cluster density vs. inter-cluster sparsity requires amathematical formalization. A very simple such formula is *coverage*, which measures the fraction of thetotal edge weight which is covered by (i.e., inside) clusters (see Formula 1). While, for a given clustering, coverage is a simple, yet decent measure for clustering goodness, trying to maximize it is unreasonable, as an optimal coverage value of 1 is attained by setting $C=\left\{C_{1}\right\}$ with *C = V*, i.e., using only one cluster. Among the established graph clustering algorithms that have successfully been applied to networks with similar structure in the past are algorithms that maximize the quality index modularity [20], which builds upon coverage. Roughly speaking, this index measures the goodness of a clustering (on a scale from -1 to 1) by comparing the coverage of a clustering to the expectation of this value if edges were rewired randomly. Equations (1) and (2) give the formulas for coverage and modularity, respectively:

(1) $$\mathrm{cov}\left(C\right):=\frac{\sum_{\text{intra}–\text{cluster}\;\text{edges}\;e}\omega \left(e\right)}{\sum_{e\in E}\omega \left(e\right)}$$

(2) $$\mathrm{mod}\left(C\right):=\mathrm{cov}\left(C\right)-\underset{\text{E}xp\left[\text{coverage}\right]}{\underset{︸}{\frac{1}{4{\left(\sum_{e\in E}\omega \left(e\right)\right)}^{2}}{\sum_{C\in C}\left(\sum_{v\in C}\sum_{e\sim v}\omega \left(e\right)\right)}^{2}}}$$

For the purpose of this article it is unnecessary to delve into the details of modularity, we refer the reader to [21-23] and further references therein. The rationale behind modularity is as follows. Given some clustering, a very low modularity tells us that even a purely random network would t the clustering better than the given graph, while a high modularity score means that the identified clustering captures very well how edges differ from randomness and constitute dense groups. The modularity of given clustering can be computed in linear time (linear in |V| + |E|), however, identifying a clustering with a high modularity is harder. In fact it is NP-hard to actually optimize modularity [21], thus heuristics are used, of which a localized agglomerative approach [24] is the current state of the art, which we also use in this work. (The term "NP-hard" from theoretical computer science indicates that it is highly unlikely that an algorithm with a practicable--subexponential in the size of the input--running time for large instances of this problem exists, roughly speaking, a heuristic is a strategy which does a good job, but no qualitative guarantee about its behavior can be given a priori.) We repeated our experiments with other graph clustering algorithms such as ORCA[25], which is strong on very large graphs, *minimum-cut tree *clustering [26], which is able to guarantee certain bottleneck properties of clusters, and *iterative conductance clustering *[27], which features a parameterized clustering coarseness. However, the constructed graphs are not large and we do not impose any specific requirements on the clustering, instead we intend to learn from the clustering and get an impression of the network. For these reasons, a self-contained modularity-based algorithm worked best for our purpose.

**Figure 4:**
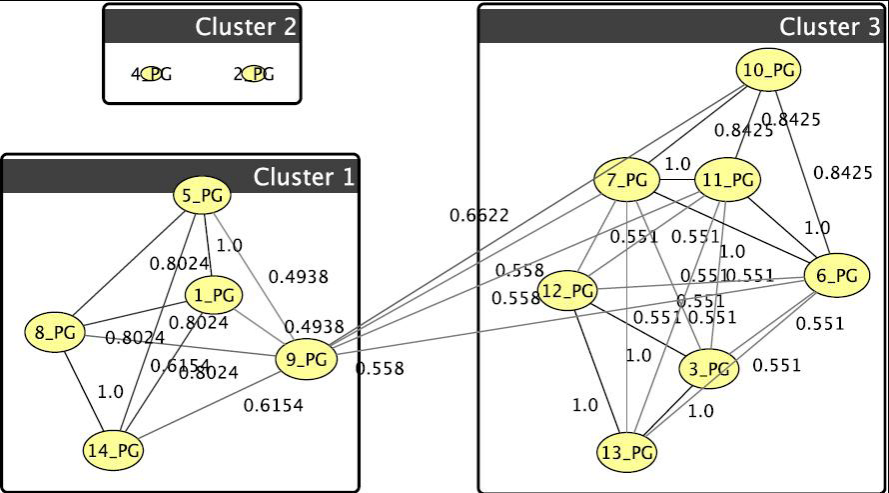
**Example of graph-clustering correlation network for phosphatidylglycerols for U87+p53/24hr**. The clustered phosphatidylglycerols wrt. treatment 6 (threshold of 0.5). Cluster 1 can also be observed in Fig. 3, cluster 3 includes most other lipids as they behave more similar than for treatment 7, only nodes 2 [(32:0)+O] and 4 [(32:1)+O] are further distant, their weak tie (0.25 < threshold) is pruned visually.

The algorithm we use [21] initializes each node as a singleton cluster, i.e., containing only this one node. Then, iteratively, each node explores its local graph neighborhood, leaves its current cluster and joins the best neighbor cluster, if such a swap yields an increase in modularity. This process usually merges many clusters. The iteration is repeated until annealed, which means that modularity can no further be improved by moving any single node to a different cluster. Resulting clusters are then abstracted to super-nodes, which means that a new graph is constructed, where each node represents a cluster in the original graph, and edges between original nodes are summarized into super-edges in the abstracted graph. In this resulting graph, the above process is repeated. Eventually, at some abstraction stage, modularity will attain its peak and no node will join any other's cluster, then the abstraction hierarchy is unfurled and the final super-nodes induce the clustering of the original graph. Figure 4 is an example clustering of treatment 6 of the phosphatidylglycerols.

### Difference Graphs and Visualization of Lipidomic Changes

For each treatment (see Table 1) and for each group of lipids we now design a correlation network as described in the previous section, and identify a graph clustering. Each clustering reveals subsets (clusters) of lipids which exhibit a very similar reaction to this treatment. Thus, roughly speaking, lipids within the same cluster can be grouped in terms of their behavior and, on the other hand, are more or less well separated from lipids in other clusters in this respect.

Generally speaking, comparing a treatment to others, e.g., a control, is of particular interest. Thus, in order to compare a first treatment *a *to a second treatment *b *of the same group of lipids, we combine two graphs and their clusterings in one new graph layout, the *difference graph*, as follows. *a*'s clustering is represented by boxes, such that each box constitutes a cluster, and *b*'s clustering by node colors, i.e., subsets of nodes with the same color constitute clusters. Comparing these clusterings yields many insights, which are immediately observable in a readable layout. It shows us how differently the two treatments "correlated the lipids". A cluster of lipids that react almost identically wrt. *a *might be split into different clusters in *b*'s clustering, which strongly indicates that the split apart nodes (i.e., lipids) share a property that makes them sensitive to treatment *b*. This selectively leads us back to specific places in the raw data, where we now know what to look for. In particular, this might even point to some biochemical feature of these lipids, the relevance of which has not yet been known. We will demonstrate this in the illustration below.

As a side note, there is some literature on quantitatively comparing clusterings of graphs or partitions of data points. A good overview of this field, with an emphasis on the peculiarities of graph clusterings, is given in [28]. A multitude of difference measures for clusterings exists, with perhaps the most prominent one being the *Rand *measure [29], which is the fraction of node pairs, which are co-classified in the same way by both clusterings, i.e., twice together or twice separated. Technically speaking, for two clusterings $C_{1}$ and $C_{2}$ of graph *G *= (*V*, *E*) set $n_{\text{tog}}{}_{.}\;=\;|\{\{u,\;v\}\in {(}_{2}^{V})|\exists C_{1}\in C_{1},\;C_{2}\in C_{2}$ such that *u*, *v *∈ *C*_1 _and *u*, *v *∈ *C*_2_}|, and set *n*_sep. _analogously for *u*, *v *separated, then $Rand\;\;(C_{1},\;C_{2})=(n_{\text{tog}}{}_{.}\;+n_{\text{sep}}{}_{.})/|{(}_{2}^{V})\}|.$. Using such a measure of comparison in our application would give a vague impression about how much treatments differ in terms of correlation of lipid reaction without looking at the graphs or knowing how the difference manifests. Using any such measure requires care and experience as they are prone to biases introduced by the size of a network and the coarseness of the clusterings. Since, additionally, we clearly focus on *qualitative *differences, which, for small graphs such as those in our application, our figures give a good impression about, we do not use difference measures.

Since the edge structure of the two compared graphs are already transferred into clusterings, we have the edges of the new difference graph represent more pressing information. the weight of edge {*u*, *v*} in a difference graph is its weight wrt. *a minus *its weight wrt. *b*. The crucial observation is as follows. This weight shows how treatments *a *and *b *differ in terms of the behavior of *u *and *v*; if this weight is close to zero, then switching from *a *to *b *does not discriminate *u *and *v*, if it is positive (negative), then *u *and *v *correlate stronger (weaker) wrt. treatment *a *than wrt. *b*. We color edges on a linear HSV-scale (from blue = cold = small difference to red = hot = large difference) by the absolute value of this new edge weight. Thus, edge colors indicate whether switching between treatment *a *and *b *discriminates nodes. However, in order not to overburden the drawing, colors encode only the absolute difference between the *a *and *b*'s correlations; this hints at where to look for interesting behavior, and detailed quantities can then be read o edge annotations or the raw data. Furthermore, we size nodes proportionally to sum of their incident absolute edge weights. Thus, in a difference-graph for *a *and *b*, a node is large if it differs strongly in its correlations to other nodes wrt. the two treatments, there should thus be several heavy (i.e., warm-colored) incident edges on such a node. Small nodes roughly preserve their correlation behavior in both treatments.

#### Illustration of the Approach

**Figure 5:**
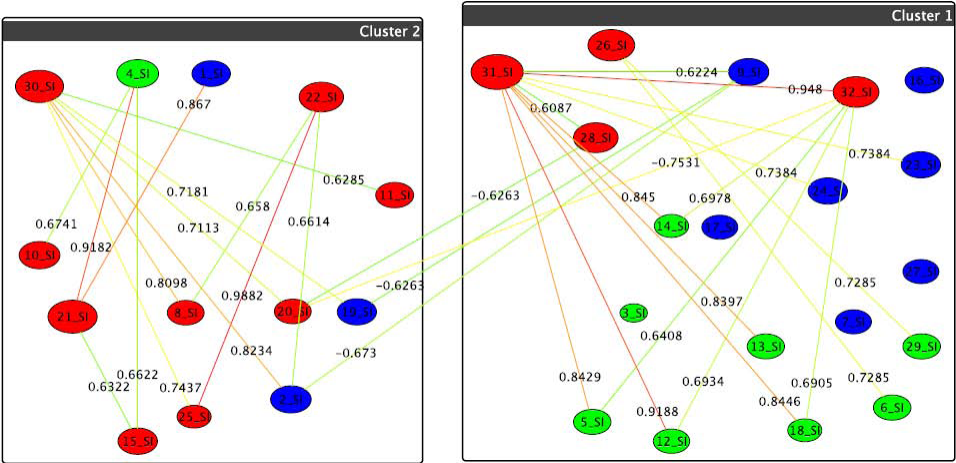
**Graph-clustering correlation network for gangliosides showing the difference between U87+DI312/24hr+SN38/24hr and U87+p53/24hr+SN38/24hr**. Correlation network resulting from graph-clustering data analysis for a correlation threshold > 0.6 for gangliosides showing the difference between the first two treatments (i.e., edge weights = cor(*T*1) - cor(*T*2)). The clustering for *T*1 (boxes) automatically divides into small-value (cluster 2) and high-value lipids (cluster 1), see Table 1. *T*2's clustering (colors) distinguishes further: high values (green nodes), intermed. values (blue nodes), and very small values (red nodes). Thus, red nodes in the right hand box show the strongest decrease when switching to T2. In particular, *v*_31 _= GD1 (d18:1/24:0) and *v*_32 _= GD1 (d18:1/24:1) show the highest change. in T1 they react similarly as several green nodes (e.g., v_5 _= GM3 (d18:1/16:1)), which they do not do for T2, as evident from the many heavy-difference edges incident to nodes *v*_32 _and *v*_31_. Also significant are *v*_30 _= GD1 (d18:1/23:1), *v*_22 _= GM1b (d18:1/23:0) and *v*_21 _= GM1b (d18:1/22:0), which, by their very small values in T2 set themselves apart from many others (heavy edges).

**Figure 6:**
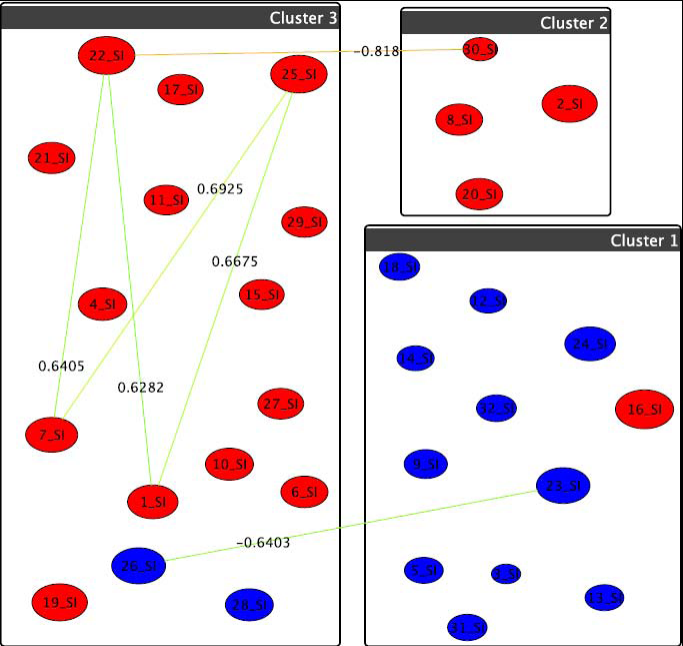
**Graph-clustering correlation network for gangliosides showing the difference between U87+SN38/24hr+DI312/24hr and U87+SN38/24hr+p53/24hr**. Correlation network resulting from graph-clustering data analysis for a correlation threshold > 0.6 for gangliosides showing no significant changes between the treatments U87+SN38/24hr+DI312/24hr (T3, boxes) versus U87+SN38/24hr+p53/24hr (T4, colors). Here, *v*_16 _= GM2a (d18:1/24:0) leaves the box of blue nodes in T4, as T4 discriminates it from the blue nodes' values. Despite of low differences in correlation between T3 and T4, in T3 (boxes) there are three different clusters induced by groups of similarly behaving lipids, and only two for T4.]

**Figure 7:**
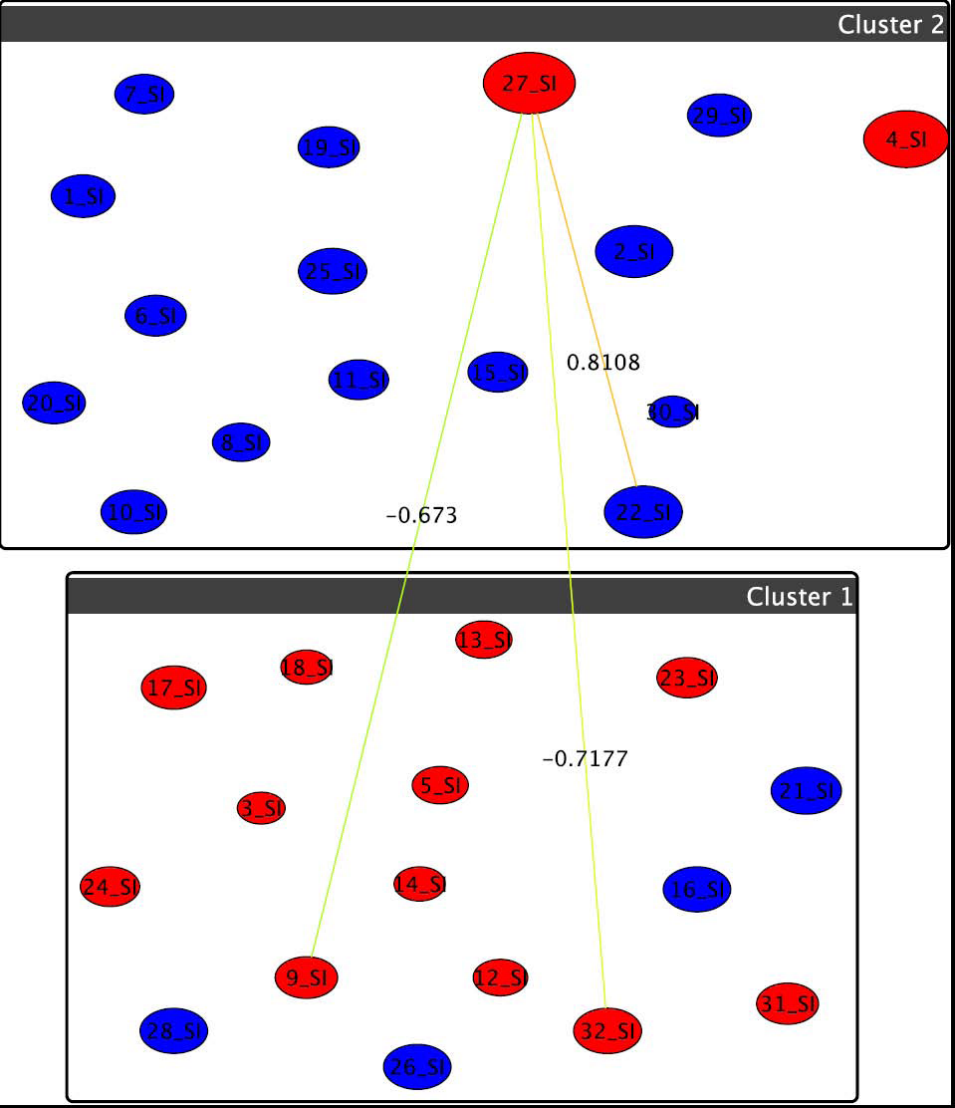
**Graph-clustering correlation network for gangliosides showing the difference between U87+DI312/24hr and U87+p53/24hr**. Correlation network resulting from graph-clustering data analysis for a correlation threshold > 0.6 for gangliosides showing no significant changes between the treatments U87+DI312/24hr (T5, boxes) versus U87+p53/24hr (T6, colors). Most notably, the size of *v*_27 _= GD1 (d18:1/16:0) and *v*_4 _= GM3 (d18:1/16:0)+O show their deviation from the behavior of the other nodes in the upper cluster, for T6, the two move to the red cluster. Most others do not di er heavily in their correlations.

Our approach is illustrated by computing the graph-correlation networks resulting from gene therapy for gangliosides, phosphatidylinositol and phosphatidylglycerol. In the following we illustrate what can be concluded from the networks constructed from these different groups of lipids, as shown in their respective figures. Please note that we do not predetermine the numbers of clusters, thus different treatments can very well yield different numbers of clusters, this simply reflects that the numbers and sizes of lipids with similar behavior depend on which treatment is used. We exemplarily show the resulting correlation networks for the group of gangliosides from Figure 1 for the strong difference of the first two treatments from Table 1 and Figure 5. In Figure 5, the result of the control treatment one is summarized by the box-clustering, and the treatment two (p53), is depicted by node colors. A first observation is that the control yields only two clusters of lipids (boxes), and adding p53 yields three (colors), thus, treatment two discriminates the reaction of lipids more finely. The red cluster is striking as it contains many large nodes, i.e., lipids, which differ strongly in their correlations wrt. the two treatments. The fan-out degree of every single lipid component in these graphs shows us the modulation degree with a given treatment. The hot (reddish) edges incident to the largest nodes show where these differences manifest most strongly, i.e., which other nodes are discriminated by such a node (and its cluster) by moving from treatment one to treatment two. Looking up the respective lipids in Figure 1, the graphical clustering thus confirms the experimental results by showing that the long chain gangliosides go down after p53 treatment. In a similar manner we can use our graphs of other groups of lipids and treatments for pointers to behavioral phenomena. For the ratio of the third and fourth treatment combinations and the fifth and sixth, respectively, from Table 1, we do not observe any relevant changes as shown in Figure 6 and 7.

**Figure 8:**
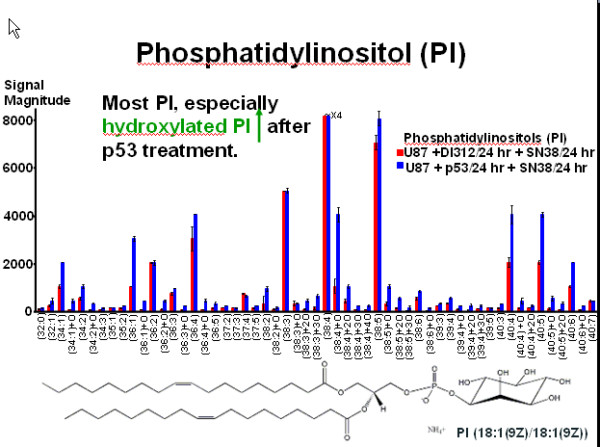
**Phosphatidylinositol profiles for the two treatments U87+DI312/24hr+SN38/24hr and U87+p53/24hr+SN38/24hr**. Phosphatidylinositol (PI) profiles for the polar lipid fraction of extracts of U87 cells following the first two treatments. The effect of treatment with wild-type wt p53, which increases the abundance of some PI, is most pronounced for the hydroxylated PIs.

**Figure 9:**
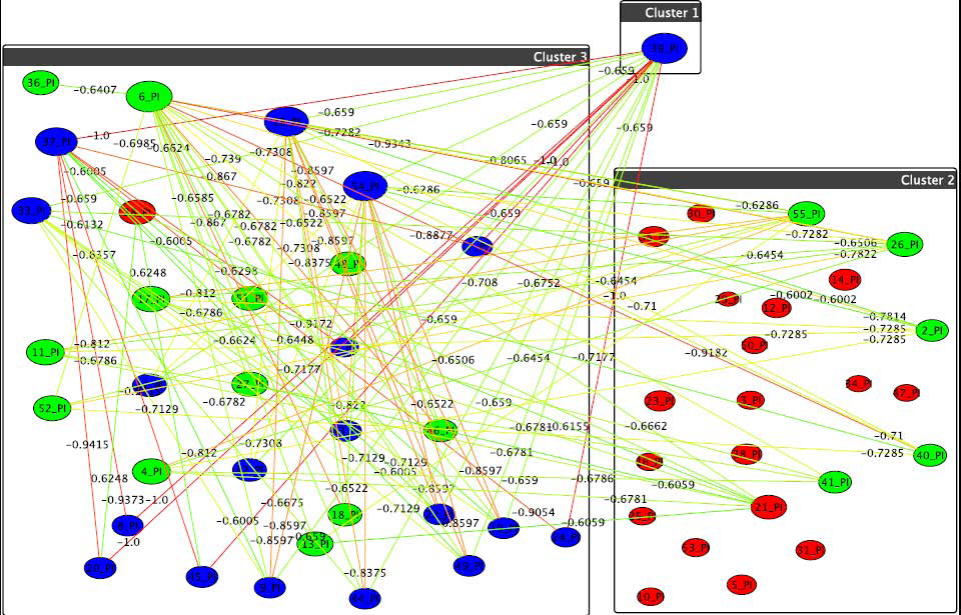
**Graph-clustering correlation network for phosphatidylinositol showing the difference between U87+DI312/24hr+SN38/24hr and U87+p53/24hr+SN38/24hr**. Correlation network resulting from graph-clustering data analysis for a correlation threshold > 0.6 for PIs showing the difference between the first two treatments. Despite of the visual clutter, we here retain threshold for the sake of comparability to the above figures. Quite obviously, T1 and T2 heavily differ, with almost 7% of all pairwise differences above 0.6. Red nodes are mostly small, which indicates little discrimination between T1 and T2 for these nodes, which in the raw data correspond to lipids with high values. Quite noticeable, *v*_39 _= (38:6)+O shows the highest increase in T2 simply because its value is 0 in T1 but 100 in T2 while *v*_6 _= (34:2)+O, *v*_15 _= (36:3)+O, *v*_37 _= (38:5)+3O and *v*_54 _= (40:6)+O are also significant.

For the phosphatidylinositol (PI), we see in Figure 8 the experimental outcome for the first two treatments showing an abundance increase of hydroxilated PIs. We can also see that the hydroxylated PI go up after p53 treatment as depicted from the difference network between the first two treatments in Figure 9. The highest increase from zero to one hundred for (38:6)+O as shown in the experimental results is represented in the network as a distinct separate cluster. There are no significant changes between the treatments U87+SN38/24hr+DI312/24hr versus U87+SN38/24hr+p53/24hr and U87+DI312/24hr versus U87+p53/24hr.

**Figure 10:**
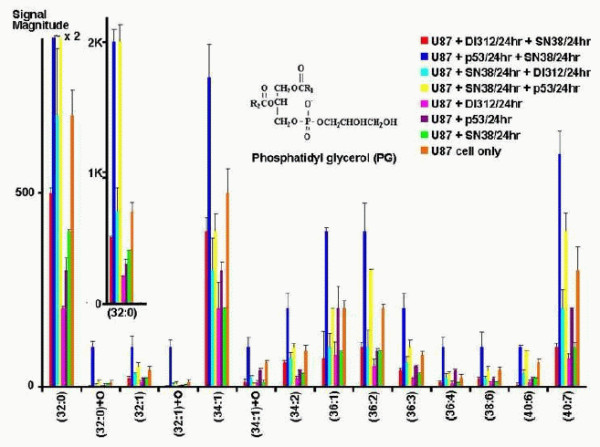
**Phosphatidylglycerol profiles for all seven treatments**. Phosphatidylglycerol (PG) profiles in U87 cells following different gene and/or chemotherapy treatments.

**Figure 11:**
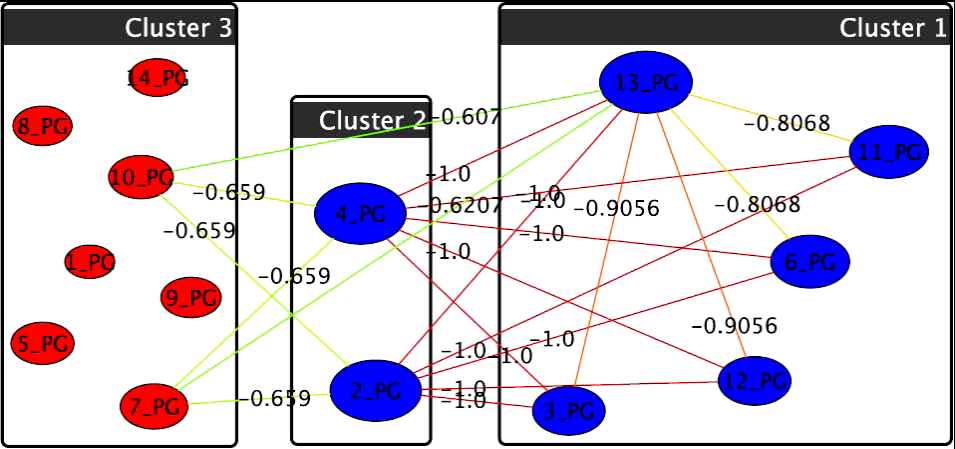
**Graph-clustering correlation network for phosphatidylglycerol showing the difference between U87+DI312/24hr+SN38/24hr and U87+p53/24hr+SN38/24hr**. Correlation network with threshold 0.6 for phosphatidylglycerols for T1 and T2. Very clearly *v*_2 _= (32:0)+0 and *v*_4 _= (32:1)+0 stand out. in T2 (colors) they abruptly behave as Cluster 1, *v*_13 _= (40:6) parallels this, but much weaker. They went from close to 0 for T1 to 100 in T2. Since the left hand nodes retain their cluster they hardly differ in T1 and T2.

The biochemical observations are again confirmed by our analysis for the phosphatidylglycerol (PG) profiles. The results for the first two treatments only are visualized in Figure 10 showing an abundance increase of hydroxylated PGs. The correlation network confirming these results is shown in Figure 11. Cluster 2 includes only the two PGs (32:0)+O and (32:1)+O showing an increase in signal magnitude from 0 for the first treatment to 100 for the second one. There are again as with the PIs, no significant changes between the treatments U87+SN38/24hr+DI312/24hr versus U87+SN38/24hr+p53/24hr and U87+DI312/24hr versus U87+p53/24hr.

We want to emphasize - based on the achieved results - that graph clustering networks compared to traditional correlation networks represent a unique "fingerprint" in lipidomics by revealing analytic properties while displaying the graph structure. This term from the field of analytic visualizations signifies a superficial drawing on the one hand, as it masks or even ignores some aspects of the raw data, however, on the other hand, it implies that prominent and potentially unknown features of the data become visible in an easily recognizable way. The above investigations show how observations made with the help of our clusterings coincide with and corroborate findings and interpretations based on expert knowledge. Graph clustering and visualizations certainly cannot replace traditional analysis, but they can be a powerful supporting tool.

## Conclusions

In this paper, we analyzed and interpreted lipidomic data sets acquired by high-throughput measurements such as nano liquid chromatography (nano-LC) separation followed by detection with high mass accuracy and high mass-resolving power 14.5 FT-ICR MS. The samples were taken from U87 cell line treated under seven different treatments. We hypothesize that the observed correlations between the gangliosides concentrations are a result of the underlying lipidomic reaction network. Thus, lipidomic networks in glioblastoma represent highly dynamical processes continuously changing under the influence of fluctuations. These induce a specific pattern of correlations and are measured in the experiment as a result of network propagation.

As seen in plant metabolism [16], the determined pair-wise correlations represent a snapshot or "finger print" of the biochemical state of the glioblastoma cell at a given point in time. We introduced a novel concept in correlation network, the so-called graph clustering approach, which results in finding not only the correlations but also the clusters in these networks providing thus based on the concept of modularity a better visualization and analysis in lipidomic data exploration.

The simulations based on the novel theoretical concept have confirmed the experimental findings: a down-regulation for gangliosides and an up-regulation for sulfatides and phosphatidylinositol. Furthermore, we could quantize the regulations across the whole lipidom showing additionally that hydroxylated phosphatidylglycerol is also up-regulated. Among the seven possible treatments, we showed that most visible changes leading to apoptosis can be found in combination with adenovirus therapy.

In summary, this paper revealed in an unique way the "fingerprints" in lipidomics showing us how gene therapy (adeno virus) with wild type p53 followed by chemotherapy with the topoisomerase inhibitor SN38 induces 95% apoptosis (cell death).

## Methods

### Data Acquisition and Analysis

The lipid analysis method has been previously described in [11], an abbreviated description of the method is outlined below.

#### Cell culture

The glioma cell line U87 MG (ATCC #HTB-14) was grown in the presence of DMEM-F12 media supplemented with 10% FBS (Cell Gro, Mediatech, Herndon, VA) in a humidified CO_2 _incubator at 5% CO_2_. Cell cultures were grown in 150-mm dishes to 90% confluency.

#### Treatment of cells

Cells (~2 × 10^6^) were treated with adenoviruses (therapeutic Ad-p53 or DI312 control adenovirus vector) or cytotoxic chemotherapy (SN-38), either alone, in combination or in different sequences. Cell cultures were treated for 24 h with SN-38 at a final concentration of 0.1 *μ*M (stock solution of 10 mM).

Cell cultures were also treated with either control virus DI312 or test virus that contained wild-type p53 gene inserted in the E1 region of the adenovirus vector (Ad-p53) at 1:100 MOI (multiplicity of infection) from a stock virus titered at 2.8 × 10^11 ^pfu (plaque-forming units). Cell cultures that were treated with a combination of drug and virus included a total incubation period of 48 h allowing 24 h for each agent. Cells were washed three times with room-temperature phosphate-buffered saline (PBS) between treatments. Prior to viral infection, the cells were placed in serum-free media for 1 hour to ensure adequate absorption of virus to the cells. These different conditions are listed in Table 1.

#### Polar lipid extraction

Cells (ca. 2 × 10^6^) were extracted by the addition of methanol:chloroform 1:1 and sonicated for 30 min. The extract was incubated overnight at 48°C in order to optimize GSL yields. After centrifugation, the supernatant was collected and partitioned with additional chloroform and H_2_O. The upper layer was collected and dried. Approximately $\frac{1}{50}\;\text{th}$ of the total extract was consumed per LC/MS experiment.

#### nLC/MS

The lipids were reconstituted in 80% methanol (aq) with the addition of 10 mM NH_4_OAc and separated by nano-liquid chromatography (Eksigent, Dublin, CA) in a self-packed 80 mm × 50 micron phenyl-hexyl column. The gradient was $\frac{15\%}{85\%}$ to $\frac{2\%}{98\%}\;\frac{A}{B}$ during 4 min (Solvent A. 98% H_2_O, 2% methanol, and 10 mM NH_4_OAc; B: 98% methanol, 2% H_2_O, 10 mM NH_4_OAc) with a flow rate of 400 nL/min. LC effluent was analyzed on-line by negative-ion micro-electrospray and a modified hybrid linear ion trap FT-ICR MS equipped with a 14.5 T magnet [12]. Parent ion mass spectra were collected at high mass resolving power (*m*/Δ*m*_50% _= 200,000 at *m*/*z *400, in which Δ*m*_50% _is spectral peak full width at half-maximum peak height) and acquisition rate (> 1 Hz). The instrument was calibrated with a mixture (ESI calibration solution, Thermo Fisher, Waltham, MA) based on the quadrupolar trapping potential approximation [30,31]. Typical broadband external calibration mass accuracy is better than 500 ppb. Data-dependent MS/MS was performed in the linear ion trap (CID, collisional induced dissociation) during collection of the ICR time-domain data [11]. Lipids were identified based on accurate mass and CID MS/MS tandem mass spectra in the LTQ. We compared tandem mass spectra to those in the literature as well as collected tandem mass spectra of lipid standards.

#### Comparative Lipid Quantitation

Comparative lipid quantitation was performed by a Semi-quantitative method based on the relative ion signal magnitude detected in FT-ICR MS of specific lipid species (glycolipid and phospholipid) for control vs. treated samples (transfectants). FT-ICR mass spectral signal magnitude is directly proportional to the number of ions of that mass-to-charge ratio [32]. Lipids were extracted, separated and analyzed under identical conditions. The complexity of the spectra per scan is simplified after nLC separation. The lipid species were well separated based on their polar head groups as well as ceramide or acyl backbones. Thus the competition for charge in the electrospray process is minimized as well as dynamic range issues in detection.

### Preprocessing

Although the measurement domain already is ${ℛ}_{+}^{\prime }$, the data has a strongly skew distribution, such that any linear scaling function lets very large values overshadow important distinctions among smaller values. For two measurements *m_u _*and *m_v _*we thus use the logarithm inside the common correlation index $\text{cor}\left(m_{u},m_{v}\right)=\frac{\log\left(1+\min\{m_{u},m_{v}\}\right)}{\log\left(1+\max\{m_{u},m_{v}\}\right)}$ (we define cor(0, 0) := 0) which is a symmetric two-variate function with codomain [0, 1]. However, preliminary models suggested this function to be too non-disciminative; we found that a cubic exponent yielding *ω*(*u*, *v*) = (cor(*m_u_*, *m_v_*))^3 ^enhances the resolution, with values well spread out in the interval [0, 1]. Note that *ω *can easily incorporate any additional data on the relationship of lipids, e.g., measurements on lipid signaling.

## Authors' contributions

RG developed the graph clustering methodology, performed data analysis and drafted the manuscript. AMB developed the lipid informatics methodology and drafted the manuscript. DW developed the graph clustering methodology. HH and MRE performed the experiments with nLC/MS and drafted the manuscript. CAC initiated the study and performed data analysis. All authors read and approved the final manuscript.
